# Medulloblastoma Presenting as Severe Headache during Pregnancy: A Case Report and Review of the Literature

**DOI:** 10.3390/medicina58010127

**Published:** 2022-01-14

**Authors:** Francesca Gabriela Paslaru, Anca Maria Panaitescu, Elena Nestian, George Iancu, Alina Veduta, Alexandru Catalin Paslaru, Lucian Gheorghe Pop, Radu Mircea Gorgan

**Affiliations:** 1 Neurosurgical Department, Bagdasar-Arseni Clinical Emergency Hospital, 041915 Bucharest, Romania; francesca.paslaru@gmail.com (F.G.P.); nestian.elen0@gmail.com (E.N.); radu.gorgan@umfcd.ro (R.M.G.); 2 Department of Neurosurgery, Carol Davila University of Medicine and Pharmacy, 020021 Bucharest, Romania; 3 Department of Obstetrics and Gynecology, Filantropia Clinical Hospital, 011132 Bucharest, Romania; george.iancu@umfcd.ro (G.I.); alina.veduta@gmail.com (A.V.); 4 Department of Obstetrics and Gynecology, Carol Davila University of Medicine and Pharmacy, 020021 Bucharest, Romania; 5 Department of Physiology, Carol Davila University of Medicine and Pharmacy, 020021 Bucharest, Romania; catalin.paslaru@umfcd.ro; 6 Dr. Victor Gomoiu Children’s Clinical Hospital, 020021 Bucharest, Romania; 7 Department of Obstetrics and Gynecology, National Institute of Mother and Child Care Alessandrescu-Rusescu, 020395 Bucharest, Romania; popluciangh@icloud.com

**Keywords:** medulloblastoma, brain tumor, pregnancy, headache

## Abstract

Headache is a common complaint during pregnancy and the puerperium. The differentiation between a benign headache and a headache that has an underlying more endangering cause, such as an intracranial tumor, can be difficult and often requires diagnostic procedures and brain imaging techniques. We report the case of an 18-year-old female patient who developed clinical symptoms—persistent headache followed by neurological deficit—in the last part of her pregnancy. A medulloblastoma (MB) was diagnosed and treated after delivery. We review 11 other cases of MB in pregnancy reported in the literature. The most common clinical manifestation at diagnosis was headache followed by neurological deficits. We discuss the association of brain tumor growth with physiological changes during pregnancy. We conclude that clinical features of intracranial tumors can be misinterpreted as pregnancy-related symptoms and should not be dismissed.

## 1. Introduction

Medulloblastoma (MB) is a primitive neuroectodermal tumor, the most common central nervous system (CNS) tumor in the pediatric population [1], but a highly uncommon occurrence in adult patients, accounting for less than 1% of all intracranial tumors [2]. Clinical signs and symptoms of MB are usually determined by increased intracranial pressure, obstruction of cerebrospinal fluid flow, and cerebellar dysfunction. Cranial nerve dysfunction becomes apparent in later stages when the tumor compresses or infiltrates the brainstem [3]. Diagnosing an MB during pregnancy or in the immediate postpartum period is exceedingly rare and further complicated by the resemblance of the clinical features to the usual complaints of pregnant patients. Due to the low prevalence of CNS tumors in the pregnant and postpartum population, no specific management guidelines are published; most of the evidence comes from individual case reports or small series of reports.

In this review, we report the case of MB in a young female patient who developed clinical symptoms in the last part of her pregnancy. We review the literature for other reported cases and discuss the suspected pathogenic mechanisms of embryonal tumor growth in pregnant patients.

## 2. Case Presentation

An 18-year-old woman was admitted to the neurosurgical department one week postpartum, with a history of intense occipital headache beginning several weeks prior to the spontaneous delivery. Her history was previously dismissed as an “usual” pregnancy symptom. During the postpartum period, she developed nausea, vomiting, and a progressively worsening headache. The patient had had an otherwise low-risk, uneventful pregnancy, and preeclampsia was ruled out.

**Figure 1:**
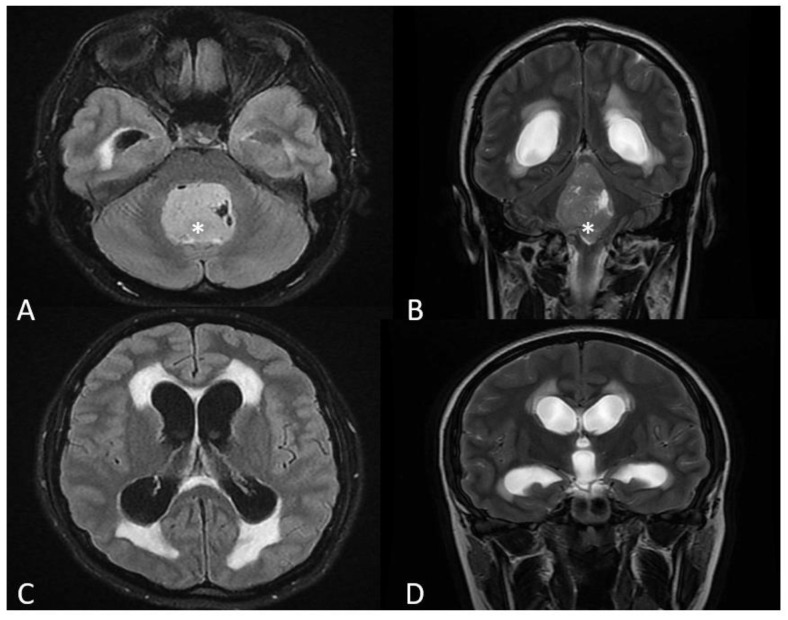
MRI scan, axial section, FLAIR sequence (**A**) and coronal section, T2 sequence (**B**), showing a large intraventricular tumor, located in the 4th ventricle. Enlargement of the lateral and third ventricles and transependimar resorption (**C**,**D**).

On physical examination, we noticed left congenital optic atrophy, a slight impairment of the left-hand coordination, and horizontal nystagmus. Optic fundoscopy showed right eye papillary edema. Breastfeeding was stopped. A gadolinium-enhanced MRI (magnetic resonance imaging) scan revealed a 33.5/33.3/48.7 mm intraventricular tumor, located in the 4th ventricle, compressing the brainstem and the cerebellum (Figure 1A,B), with cerebellar tonsillar herniation through the foramen magnum. The lesion showed inhomogeneous gadolinium enhancement. Secondary triventricular obstructive hydrocephalus was also noted (Figure 1C,D). The imagistic aspect was suggestive of MB.

Two days after admission to the hospital, the patient started complaining of difficulty in swallowing, and we noted bilateral impaired pharyngeal reflexes and left side deviation of the uvula. Considering the progressive nature of the neurological symptoms and the imagistic aspect, rapid neurosurgical treatment was deemed necessary. We performed a midline suboccipital craniotomy and direct transvermian approach, obtaining a gross total resection of the tumor, thus removing the cause of the cerebrospinal fluid obstruction [4]. Concurrently, we administered 8 mg dexamethasone per day to reduce perilesional vasogenic edema. 

**Figure 2:**
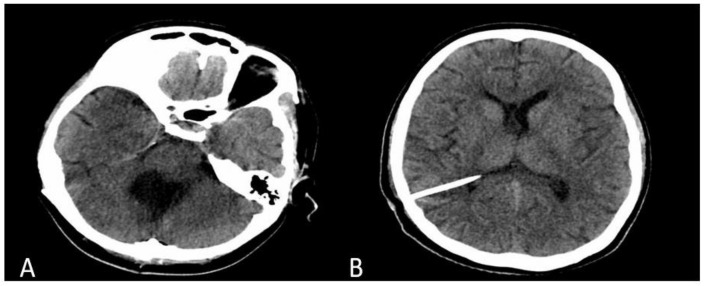
Non-contrast enhanced CT scan showing the resection cavity in the posterior fossa (**A**) and intraventricular catheter and normal-sized lateral ventricles (**B**).

Tissue pieces were sent for histopathological examination, which confirmed the initial diagnosis, further classifying the tumor as a classic variant of MB as per the revised WHO classification of tumors of the central nervous system (CNS) published in 2021 [5]. An initial control computed tomography (CT) scan showed complete resection of the 4th ventricular tumor, with no signs of postoperative hemorrhage, and reduced dimensions of the lateral and 3rd ventricles. Despite the normal early postoperative imagistic aspect (Figure 2A), the patient required a ventriculo-peritoneal shunt at 15 days after surgery. A second postoperative CT scan showed a decrease in size of the lateral and 3rd ventricles (Figure 2B) and the patient recovered well with slow remission of the neurological symptoms. She was referred to oncology, for adjuvant chemotherapy (Packer protocol—vincristine, cisplatin, lomustine) and radiotherapy (53.2 Gy in fractions of 1.6 Gy daily, five times weekly and a local dose up to 54–55.8 Gy to the posterior fossa) [4]. At 6 months follow-up, the patient was in a good condition, with only mild ataxia and dysarthria, but still under the care of the oncological and neurosurgical team.

## 3. Discussion

We present a case where in pregnancy and postpartum, severe headache was caused by a rare intracranial tumor—a medulloblastoma. Headache is a common complaint during pregnancy and puerperium, being reported at least once by 35% of all pregnant women [6]. The differentiation between a benign headache and an underlying more endangering disorder can be difficult and often requires diagnostic procedures, such as brain imaging techniques. Although primary headaches, such as a tension headache or a migraine, account for most cases [7], more than one-third of the acute headaches in pregnant patients are secondary to other causes [8,9].

When considering an imaging modality, one should take into account the risk of adverse effects to the developing fetus. To reduce unnecessary investigations, in 2016 Mitsikostas et al. published the European Headache Federation consensus on technical investigation for primary headache disorders, emphasizing a series of “red flags,” clinical features that would represent a warning of possible underlying disorders (Table 1) [10]. The most common neurological disorders leading to secondary headache during pregnancy include hypertension, infections, cerebral deep vein thrombosis, brain ischemia, and intracranial hemorrhage [8,11].

In our case, the sudden onset of headache and the progressive worsening may have been the first clinical features suggesting a secondary cause headache, followed by the progressive neurological deficits diagnosed postpartum. Although secondary headaches during pregnancy are not uncommon, a diagnosis of a brain tumor in a pregnant patient can still be shocking, given its rarity. Because of the low prevalence of CNS tumors during pregnancy, to our knowledge no specific management guidelines have been published, and most of the evidence comes from individual case reports or small series of reports. We identified 10 articles in the literature, comprising 11 cases of MB diagnosed during pregnancy (Table 2) [12,13,14,15,16,17,18,19,20,21]. Medulloblastoma was diagnosed in rather young pregnant women. The most common complaint at diagnosis during pregnancy was headache followed by neurological deficits. There was one maternal death due to MB. Medulloblastoma was diagnosed in the second and third trimester of pregnancy in most cases and delivery was arranged by caesarean section in most of the cases remote from term. Six of the babies included in our study of the literature were delivered preterm. Two cases that were diagnosed early in pregnancy (first trimester or early second trimester) underwent termination of pregnancy to allow surgical and oncological treatment. Gross removal of the tumor was intended in all cases, and after delivery seven women underwent oncological treatment.

Regarding the use of CT and MR imaging techniques in pregnant or lactating women, guidelines recommend an individualized patient-based approach considering the risk-to-benefit ratio. Magnetic imaging is generally considered safe during pregnancy; however, gadolinium administration is best avoided if possible. A CT scan for neuroimaging of the brain is considered to expose the fetus to an insignificant level of irradiation and recommendations are that it should not be withheld if required, irrespective of the trimester of pregnancy [22].

Pregnancy generates a series of important hormonal changes and increased levels of angiogenic and growth factors, thus influencing the growth rate of brain tumors—especially meningiomas, but also glial tumors and MB [21,23,24]. Angiogenesis has been proven to be necessary for tumor growth, and metastasis and has been correlated with prognosis and survival for many cancer types, including MB [25,26]. The cerebellum has been ignored as a site of steroid hormone action for a long time [27]. Hedges et al. published evidence for defining the cerebellum as a target of estradiol signaling, further proving the importance of estrogen-driven pathways in the development of the cerebellum, synaptic neurotransmission modulation, and cerebellar diseases, such as cancer [28]. Estrogens exert their effect through the two estrogen receptors, estrogen receptor α and β (ER α and ER β), encoded by separate genes [27]. In contrast to the α subtype of estrogen receptor, whose role in estrogen dependent neoplasms (breast, uterus, and prostate cancer) is well established, the potential involvement of the β subtype ER in CNS neoplasms is under investigation. Studies have shown that cerebellar differentiating granule cell precursors (GCPs) transiently express high levels of estrogen receptors β (ERβ) and that their proliferation and viability is regulated by low levels of 17 β-estradiol (E2) [29,30]. Therefore, cellular response to estrogen receptor stimulation might explain the appearance and growth of MB during pregnancy, when high E2 levels are registered [21].

It has been documented that ERβ is expressed by both precursor and mature granule cells, as well as MB cells, and that estrogen can influence proliferation and migration not only in healthy GCPs, but also in MB cells, by altering gene expression [25,27]. ERβ-mediated increases in insulin-like growth factor receptor 1 expression and activity accounted for cytoprotective mechanisms, resulting in decreased apoptosis [28]. Consequently, pharmacologic blockade of ERβ in cell cultures and xenograph models of MB has been shown to inhibit tumoral migration and growth, indicating that estrogen antagonists could be used as adjuvant therapy to a current cytotoxic regimen to cure this malignancy [25,27,28].

## 4. Conclusions

The case we present illustrates the fact that, while common fetal and maternal conditions can be systematically addressed by prenatal care [31], severe rare diseases can sometimes unpredictably develop during pregnancy, even in the lowest risk patients.

Due to the low prevalence of medulloblastoma in pregnant patients, there is little evidence regarding the pathogenesis and treatment strategies. Clinical features can be misinterpreted as pregnancy symptoms. Gross total resection is the goal of surgical intervention, followed by adjuvant radiotherapy and chemotherapy after delivery. Obstetricians caring for pregnant women should always keep in mind that some symptoms of pregnancy may represent potential “red flags,” and should be taken into consideration.

## Figures and Tables

**Table 1:** Approach to investigating headaches in pregnant or postpartum patients.

Step	Approach
1	Investigate for pregnancy-related complications that can manifest with headaches - hypertensive disorders of pregnancy, preeclampsia - cerebral deep vein thrombosis - intracranial hemorrhage - infections
2	Perform a basic neurologic exam
3	Investigate for “red flags” in association with headaches - history of malignancy - history of HIV or active infections - seizures - fever - previous trauma - neurological symptoms and signs - headache precipitated by physical activity or Valsalva maneuver - headache that changes with posture - headache that awakens the pregnant woman - changes in a previously stable headache pattern - new headache type or headache that takes less than 5 min to peak in severity
4	Consider options for diagnostic imaging

**Table 2:** Cases of MB in pregnant patients published in the literature. C-section: caesarean section; GTR: gross total removal; STR: subtotal removal; RxT: radiotherapy; ChT: chemotherapy.

First Author, Year	Age (Years)	GA at Diagnosis (Weeks)	Symptoms	Metastases	Mode of Delivery	Treatment
Pollack et al., 1993 [12]	21	20	Not specified	Bone marrow, placenta	C-section at 29 weeks	Not specified
Nishio et al., 1996 [13]	23	25	Headache, right hand ataxia		C-section at 33 weeks	GTR, postpartum RxT
Razak et al., 2005 [14]	24	26	Headache, diplopia, ataxia		C-section at 30 weeks	STR, RxT, ChT
Aravind et al., 2007 [15]	19	30	Headache, vomiting		Vaginally	Intended GTR
Ishak et al., 2011 [16]	34	2nd trimester	Syncope, headache, vertigo		Termination of pregnancy	Surgical resection
Kwalk et al., 2011 [17]	32	26	Not specified	Spine	C-section at 29 weeks	Intended STR, RxT, ChT
Ventura et al., 2016 [18]	28	33	Sudden death			
Sharma et al., 2013 [19]	28	30	Headache, vomiting, vertigo, diplopia		Vaginally	Intended STR
Gergawi et al., 2019 [20]	29	13	Headache, vomiting, vertigo		C-section at 32 weeks	GTR, ChT
Valazero et al., 2021 [21]	21	13	Headache, gait disturbance, right hemiparesis, dysarthria	bone marrow	C-section at 37 weeks	STR, postpartum RxT, ChT
	20	8	Headache, tonic-clonic Headache, tonic-clonic seizures		Termination of pregnancy at 13 weeks	GTR, RxT, ChT
Our case	18	3rd trimester	Headache, antepartum; headache, nausea, vomiting, left hand ataxia, horizontal nystagmus postpartum		Vaginally	GTR, RxT, ChT

## Data Availability

Not applicable.
